# Beyond the Usual Age: A Case Report on Segmental Colitis Associated With Diverticulitis in a Young Patient

**DOI:** 10.7759/cureus.80661

**Published:** 2025-03-16

**Authors:** Samantha Brophy, Nicole Hitomi, Gurvijay Bains

**Affiliations:** 1 Emergency Medicine, California Northstate University College of Medicine, Elk Grove, USA; 2 Emergency Medicine, University of the Pacific School of Health Sciences, Sacramento, USA; 3 Emergency Department, Kaiser Permanente, Modesto, USA

**Keywords:** abdominal pain, diverticulitis, diverticulitis management, diverticulitis mimic, emergency medicine, scad management, segmental colitis associated with diverticulitis

## Abstract

Diverticulitis is a common condition generally seen in older populations. It is rare, but not impossible, to see in younger patients where it can often be confused with other abdominal pathologies. Segmental colitis associated with diverticulitis (SCAD) had been previously considered a complication of diverticulitis, and only fairly recently has it been designated as its own pathology. It is typically defined as a non-specific inflammation localized to the sigmoid region of the colon, with features that may appear similar to inflammatory bowel diseases or acute uncomplicated diverticulitis. We present the case of a 22-year-old male patient who presented to the emergency department (ED) with left lower quadrant (LLQ) pain, suprapubic pain, and nausea, and was found to have SCAD on abdominal imaging. The patient improved with pain medication and antiemetics and was found to be stable for discharge home with appropriate outpatient follow-up. This case presents an unusual presentation for this pathology and brings attention to the importance of a broad differential in the ED as well as understanding management plans and similarities between disease presentations.

## Introduction

The incidence of emergency department (ED) visits is on the rise, with reports from 2019 showing that 21.8% of adults aged 18 and above had at least one or more ED visits [1]. Abdominal pain is one of the most common complaints, accounting for approximately 11,000 visits in 2020 alone, and yet there is no definitive pathway for diagnosing and managing this complaint [2,3]. Approximately 25% of patients are discharged from the ED with a diagnosis of "non-specific abdominal pain" [4]; however, it is important that we take note of the various differentials and what makes a case emergent versus non-emergent. When caught early, many causes of abdominal pain can be managed non-emergently, but if ignored, they can progress into emergent cases that require more invasive interventions and may have less than optimal outcomes for patients. As the scope of practice in emergency medicine grows and as our patient population continues to expand, we must constantly be aware of the different disease pathologies, their presentations, and their management plans. While it is rare to see certain diseases in younger patients, it does not negate the possibility. Here, we present a case of a 22-year-old male who arrived at the ED with left lower quadrant (LLQ) abdominal pain and nausea without vomiting and was found to have segmental colitis associated with diverticulitis (SCAD).

## Case presentation

A 22-year-old male with a past medical history of a single episode of diverticulitis presented to the ED for two days of abdominal pain. The pain was located in the LLQ and suprapubic region and was described as being sharp and cramping in nature and similar to his prior episode of diverticulitis. He endorsed some urinary urgency and nausea without vomiting. He denied any recent fevers, illnesses, sick contacts, dysuria, hematuria, and hematochezia. 

During the examination, the patient was noted to be tachycardic to 110, hypertensive at 160/120, and afebrile. The remaining vital signs were stable. The ED physician noted tenderness without guarding or rebound tenderness in the LLQ and suprapubic regions. The abdomen was flat and soft with no peritoneal signs. The remainder of the exam was unremarkable.

**Figure 1:**
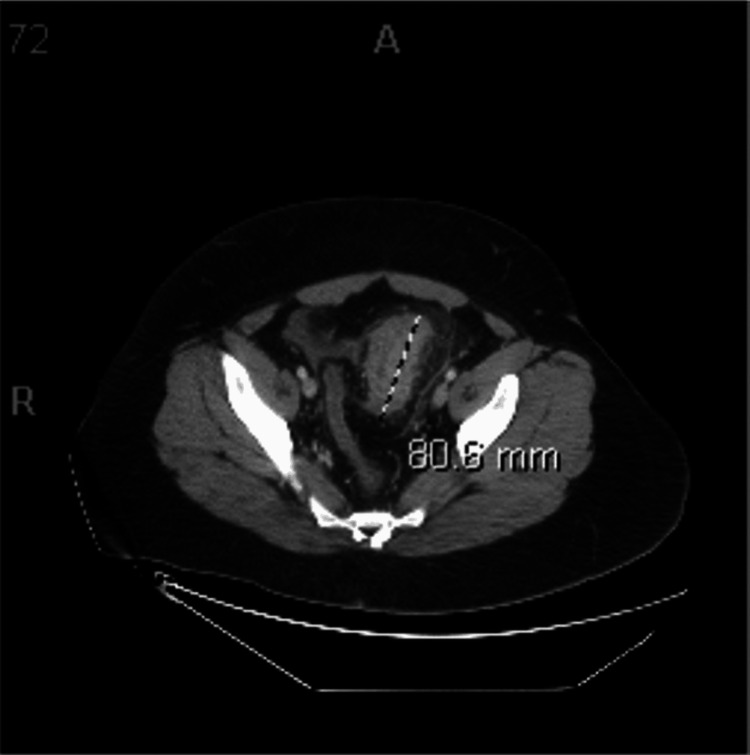
Axial abdominal CT with annotation showing the area of inflammation CT = computed tomography

**Figure 2:**
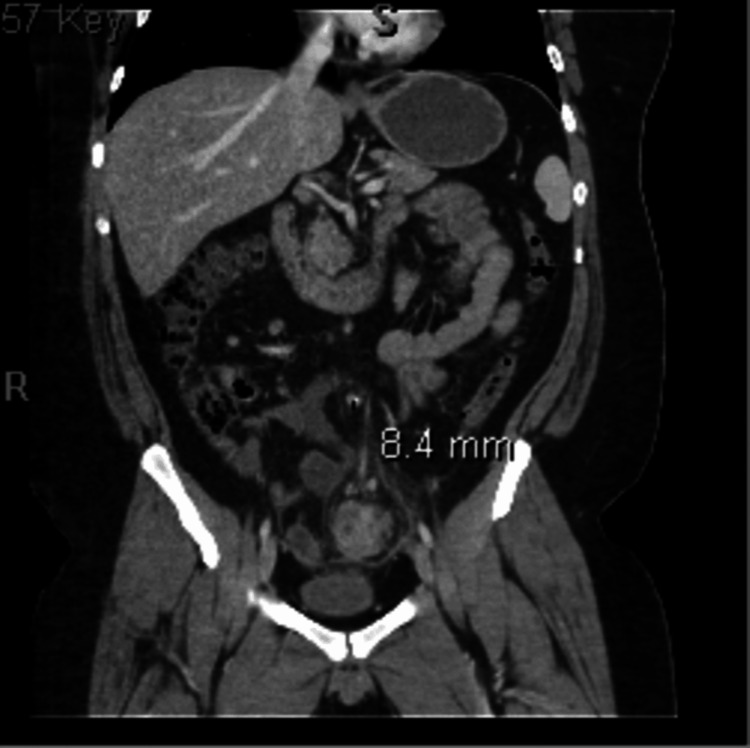
Coronal abdominal CT showing an area of inflammation near the diverticulum CT = computed tomography

During his course in the ED, he received 30 mg IM Toradol and 4 mg PO Zofran. Labs showed a white blood cell (WBC) count of 20.1, alanine aminotransferase (ALT) of 85, and bilirubin of 1.4, not significantly elevated, and with no prior labs to reference. Abdominal computed tomography (CT) was read as showing the following:

Acute inflammatory process involving the proximal and mid sigmoid colon extending over a length of 10 to 12 cm with marked circumferential wall thickening (Figure 1) and heterogeneous enhancement of the wall of the sigmoid colon with significant infiltration/inflammatory reaction extending into the sigmoid mesentery and multiple clustered small enhancing lymph nodes in the sigmoid mesentery and along the draining mesenteric vein. There are diverticula in this segment of the colon; however, this inflammatory process is not centered on a discrete inflamed diverticulum (Figure 2). The length of involvement is longer than is typically seen with acute diverticulitis. Findings may represent SCAD. A neoplastic process cannot be definitively excluded, but follow-up with a gastroenterologist will be beneficial for further classification and diagnosis. 

The on-call general surgeon did not recommend immediate surgical intervention but recommended PO antibiotics with outpatient follow-up. On re-evaluation following medication administration, the patient reported improvement in pain and nausea. Findings from imaging were discussed along with the importance of prompt follow-up with surgery and a primary care provider (PCP). The patient had an allergy to Penicillin so he was discharged with Ciprofloxacin 500 mg bid for five days and Metronidazole 500 mg tid for five days. At the time of this publication, no follow-ups or consults had been obtained by the patient.

## Discussion

Diverticular disease is defined by the presence of diverticula, which are protrusions from the colonic wall [5]. It has rapidly become a significant contributor to ED presentations and emergent surgical cases, as well as elective surgical cases. This disease comes with complications, ranging from complicated diverticulitis warranting emergent surgery to clinically mild courses of the disease [5], and accounts for approximately 600,000 visits in 2019 and trending upward [6]. The age distribution within the United States has been well-documented in older patients, with rates of occurrence rising with age. Recent studies have shown that the majority of patients presenting with diverticulitis were distributed equally from ages 40 to 80, and it was uncommon in individuals younger than 40 years [7]. Recent years have shown a trend in the positive direction for outcomes in these younger patient populations despite the increase in rates for hospitalizations [7]. Management options for uncomplicated diverticulitis are antibiotics, a liquid diet, and bowel rest with high fiber intake. Antibiotics are generally reserved for patients that meet certain criteria, including elevated white blood cell (WBC) count over 15,000/mm, C-reactive protein over 140 mg/L, the presence of fluid collection, comorbidities, and immunosuppression [8]. For complicated diverticulitis, which is defined as having a phlegmon, abscess, stricture, perforation, obstruction, or fistula, the management options are bowel rest and antibiotics with surgical consult for possible surgical intervention [9]. All complicated cases require admission, especially if abscesses are seen on abdominal imaging.

While SCAD had previously been viewed as a complication of diverticular disease, it recently was given status as its own disease pathology. While poorly understood, there have been several implicated processes such as ischemia, fecal stasis, and prolapse - pathologies that lead to chronic inflammation within the mucosa or changes within the gut flora and activity [10]. The most typical known presentation is diarrhea, LLQ cramping, abdominal pain, and hematochezia, and a prior history of diverticulitis is not a requirement to make this diagnosis [10]. SCAD has been primarily identified via endoscopy/colonoscopy during the evaluation of presenting symptoms in an attempt to rule out occult malignancies and has shown inflammation affecting the sigmoid colon with sparing of the rectum [11]. Histologically the chronic inflammation presents as a crescentic fold pattern with lymphocyte and neutrophilic infiltrates [12]. This disease process is typically seen in elderly males over the age of 50, with over 70% presenting with the symptom of rectal bleeding [13]. One of the main features of SCAD is the good response to limited treatment. 

The treatment for uncomplicated diverticulitis has long been antibiotics that target gut flora. With the classification of SCAD as its own disease as well as its ability to be self-limited with low recurrence and similar presentation to inflammatory bowel diseases (IBD), such as ulcerative colitis and Crohn’s, there have been alternative management options studied. The optimal treatments have not been well-studied, leaving the door open to different treatment potentials. Currently, treatment guidelines are to begin with Ciprofloxacin 500 mg bid for 10-14 days and Metronidazole 10 mg/kg per day for 10-14 days. For patients who don’t respond to antibiotic treatment, 5-aminosalicylic acid (5-ASA) medications can be trialed [10]. 5-ASA medications have been used to treat inflammatory bowel diseases, although usually with more long-term courses. Specific recommendations are for Mesalamine 800 mg tid for 7-10 days, and if adequate response has not been achieved in 2-4 weeks, it can be increased to 1600 mg tid [10]. For those failing both antibiotic and 5-ASA management, Prednisone 40 mg daily for one week with a gradual taper to discontinuation over the next six weeks with an alternative being Budesonide [10]. Ideally, surgical management with resection and potential colostomy is reserved only for those who have failed medical management or have relapsed shortly after treatment stops. There have been cases reported where these therapies have not worked and more aggressive management with immunosuppressives such as tumor necrosis factor (TNF)-ɑ overexpression has been beneficial [14].

In the ED, it is important to recognize SCAD as a possible differential for LLQ pain, even in patients who don’t necessarily fit the profile, as evidenced by the case presented here. The important causes of pain in a male patient that should not be missed include testicular torsion, incarcerated hernia, small/large bowel obstruction, diverticulitis, bowel perforation, and nephrolithiasis. Less emergent differentials include irritable bowel disease, colitis, constipation, malignancy, and pyelonephritis. The most common confounding differentials that would be excluded with either imaging or colonoscopy with biopsy would be diverticulitis and IBDs, like Crohn’s or ulcerative colitis. Where IBD typically affects younger patients and SCAD typically affects older patients, this is not a hard and fast rule and SCAD can still affect younger patient populations. Ulcerative colitis has inflammation that always affects the rectum, Crohn’s disease can affect every GI region, and SCAD is typically confined to the sigmoid colon region with rectal sparing [15]. The relapse rates for IBD are much higher and often require maintenance medications while SCAD is often self-limited and can potentially improve without any interventions and without relapse [15]. When comparing diverticulitis to SCAD, colonoscopy, biopsy, and imaging may help you differentiate. Imaging will show larger areas of inflammation with SCAD, not contained to a single causative diverticulum [16]. The biopsy will show chronic inflammatory infiltrate only visible during acute cases of diverticular disease, whereas SCAD will show typical crescentic lesions, glandular distortion, inflammatory infiltrate, and possible cryptic abscesses [17]. Another differentiator can be TNF-ɑ values, which have been identified as a contributor to this disease, and so will be higher in SCAD and lower in diverticular disease [15].

## Conclusions

The role of the emergency physician is to rule out emergencies, and while SCAD and diverticular disease may not always be emergent cases, it is important to understand the disease pathology to provide accurate and on-time care to patients. Recognizing the similarities and differences between the different diagnoses on your differential list can change the outcome from a patient who returns due to inadequate medical interventions and possibly needing more emergent care, to one who can be followed by an outpatient team. SCAD is fairly new in the field of medicine as its own pathology, and documenting occurrences in case reports can help us as physicians to create a better workflow and differential list in these patients. The variety of presenting symptoms makes it more difficult, so continued documentation of cases that stray from the expected presentation will benefit similar cases in the future. It is uncommon to see both diverticular disease and SCAD in a patient in their 20s, which is why this case presentation provides valuable information while providing care: understanding which consults to call, tests to order, and appropriate follow-up.
